# Medical Acute Complications of Intracerebral Hemorrhage in Young Adults

**DOI:** 10.1155/2015/357696

**Published:** 2015-02-02

**Authors:** Riku-Jaakko Koivunen, Elena Haapaniemi, Jarno Satopää, Mika Niemelä, Turgut Tatlisumak, Jukka Putaala

**Affiliations:** ^1^Department of Neurology, Helsinki University Central Hospital, Haartmaninkatu 4, P.O. Box 340, 00290 Helsinki, Finland; ^2^Department of Neurosurgery, Helsinki University Central Hospital, 00290 Helsinki, Finland

## Abstract

*Background.* Frequency and impact of medical complications on short-term mortality in young patients with intracerebral hemorrhage (ICH) have gone unstudied. *Methods.* We reviewed data of all first-ever nontraumatic ICH patients between 16 and 49 years of age treated in our hospital between January 2000 and March 2010 to identify medical complications suffered. Logistic regression adjusted for known ICH prognosticators was used to identify medical complications associated with mortality. *Results.* Among the 325 eligible patients (59% males, median age 42 [interquartile range 34–47] years), infections were discovered in 90 (28%), venous thrombotic events in 13 (4%), cardiac complications in 4 (1%), renal failure in 59 (18%), hypoglycemia in 15 (5%), hyperglycemia in 165 (51%), hyponatremia in 146 (45%), hypernatremia in 91 (28%), hypopotassemia in 104 (32%), and hyperpotassemia in 27 (8%). Adjusted for known ICH prognosticators and diabetes, the only independent complication associated with 3-month mortality was hyperglycemia (plasma glucose >8.0 mmol/L) (odds ratio: 5.90, 95% confidence interval: 2.25–15.48, *P* < 0.001). Three or more separate complications suffered also associated with increased mortality (7.76, 1.42–42.49, *P* = 0.018). *Conclusions.* Hyperglycemia is a frequent complication of ICH in young adults and is independently associated with increased mortality. However, multiple separate complications increase mortality even further.

## 1. Introduction

Nontraumatic intracerebral hemorrhage (ICH) is a major cause of morbidity and mortality worldwide caused by a variety of different etiologies and with a case fatality at 90 days of around 35% [1–3]. So far, treatment of ICH has been largely supportive with aims to prevent further brain injury and prevent and treat associated medical and neurological complications, such as hypertension, hyperglycemia, infections, increased intracranial pressure, seizures, electrolyte disturbances, and venous thrombotic events, which may add even further harmful effects to an already lethal disease [4]. Pulmonary complications, such as pneumonia and pulmonary embolism, and urinary tract infections have previously been reported to increase mortality, morbidity, and length-of-stay in hospital among patients with ICH [5–8]. Hyperglycemia has also been reported to worsen the outcome after ICH [9, 10].

The roles of medical complications have not yet been investigated among young adults with ICH in the few studies executed in young patients with ICH [11–18]. The young patients have different risk factors, etiology, and outcome, they have fewer coexisting chronic diseases than older ICH patients, and so their array of medical complications and their impact on outcome may differ as well [19]. We aimed to describe the in-hospital medical complications in young adult patients with first-ever ICH due to any cause and to study their impact on short-term mortality.

## 2. Methods

This study is based on the Helsinki ICH in the Young Study, a retrospective analysis of all the consecutive patients with a nontraumatic first-ever ICH occurring at the age of 16 to 49 years treated in the Helsinki University Central Hospital (HUCH) between January 1, 2000, and March 31, 2010 [19]. This study has been approved by institutional authorities. Consent for registration was not required by the Finnish legislation as this was a registry-based study with no patient contact.

### 2.1. Patient Selection

Eligible patients were selected out of all the patients who at any time during their hospital stay or outpatient visit had an ICD-10 (International Classification of Diseases, 10th Revision) diagnosis code of Q28.1, Q28.3, I60.8, I61, I67.3, I67.4, I67.5, I67.6, I67.7, I67.8, I67.9, I68, or I78 through screening their brain scans. Patients with ICH due to trauma or secondary to subarachnoid hemorrhage or ischemic stroke were excluded, as were patients without available brain CT or MRI scans.

### 2.2. Data Retrieval

A retrospective chart review including patient medical records, laboratory database, and imaging database was executed. All patients had been initially seen by a neurologist or a neurosurgeon. NIH Stroke Scale (NIHSS) was used to assess stroke severity. Hematoma volumes were calculated with the ABC/2 method [20]. Presence of intraventricular extension of hemorrhage (IVH) was recorded but not included in volume calculations. Hematoma location was classified as lobar, deep, infratentorial, or mixed [21]. We recorded neurosurgical hematoma evacuation operations performed. Use of nasogastric tube, intubation, tracheostomy, central venous catheter, urinary tract catheter, and antibiotic treatment was recorded. Furthermore, use of bacterial culture sample from cerebrospinal fluid, urinary tract catheter, and tracheostomy or intubation tube was recorded.

### 2.3. Medical Complications

We recorded medical complications patients suffered any time during their acute hospital stay due to ICH, including infections, venous thrombotic events, cardiac complications, renal failure, hypoglycemia, hyperglycemia, and sodium or potassium imbalances.

We considered as respiratory infections both pneumonias verified as infiltrate in chest radiograph together with fever, aural temperature >38°C, and tracheal infections verified by bacterial growth in tracheal culture in intubated or tracheostomized patients [5]. Septicemia was defined by bacterial growth in blood culture together with fever. Urinary tract infections (UTI) were registered by positive urine dipstick for nitrate or pyuria together with bacterial growth in urine sample culture. Meningitis was recorded by bacterial growth in cerebrospinal fluid sample accompanied by fever. Gastrointestinal infection was diagnosed by diarrhea or peritonitis verified in laparotomy.

Deep venous thrombosis (DVT) was recorded only when verified by ultrasound imaging of veins, and pulmonary embolism (PE) had to be verified by pulmonary CT angiography imaging. Cardiac complications included first-ever atrial fibrillation, atrial flutter, and ventricular fibrillation diagnosed by electrocardiogram (ECG) or myocardial infarction verified by ECG and elevated myocardial enzyme levels [22].

Hyperglycemia was defined as plasma glucose >8.0 mmol/L (>144 mg/dL) at any time during acute hospital ward. Hypoglycemia was defined as plasma glucose <4.0 mmol/L (72 mg/dL) [23]. Renal failure was defined as plasma creatine level >100 *μ*mol/L measured by enzymatic method [24]. Highest and lowest plasma sodium and potassium levels during hospital stay were recorded and were defined as normal by the range of values used by the Central Laboratory of Helsinki and Uusimaa Hospital District (http://www.hus.fi/en/medical-care/laboratory): 136–145 mmol/L (313–333 mg/dL) for sodium and 3.3–5.0 mmol/L (12.9–19.6 mg/dL) for potassium.

Our written institutional stroke treatment guidelines have recommended treatment of hyperglycemia with subcutaneous insulin every hour until level of plasma glucose <8.0 mmol/L is reached. Intravenous insulin is used in treatment-resistant cases. Intravenous fluids with calculated percentage of sodium and potassium are administered to patients routinely to correct sodium or potassium disturbances if present.

### 2.4. Outcome Data

The primary outcome measure was 3-month all-cause mortality. Mortality data was obtained from Statistics Finland (census date 25 June 2013). No patient was lost to follow-up. Discharge to rehabilitation center or home was recorded.

### 2.5. Statistical Analyses

Normality of continuous variables was tested. Results were analyzed in demographic subgroups of patients between 16 and 29, 30 and 39, and 40 and 49 years of age. Comparisons were done between patients receiving conservative or operative treatment. Categorical variables were compared with Chi square and Fisher's exact test. Mann-Whitney *U*-test was used to compare continuous variables with a skewed distribution between 2 and Kruskal-Wallis test between >2 groups. To study the impact of medical complications on mortality, we constructed a binary logistic regression model using complications associated with 3-month mortality in univariate analysis with adjustment for covariates known to associate with mortality in general and young ICH patients [16, 25–27]. These covariates included age, gender, hematoma volume, infratentorial hematoma location, presence of intraventricular hemorrhage, NIHSS score, and hematoma evacuation. Urinary tract infection was excluded from this analysis due to possible observational bias. Because preexisting diabetes and hyperglycemia are intercorrelated, we forced diabetes in the model. To illustrate the additive impact of multiple different complications, we also performed a multivariable analysis with number of complications (score) as a covariate instead of individual complications. Backward likelihood-ratio method was used in all regression analyses. A two-sided *P* value < 0.05 was considered significant. All analyses used SPSS 21 for Windows (IBM Inc., Armonk, NY, USA).

## 3. Results

Out of 1325 patients screened, a total of 325 patients were eligible for the study (200 males, 59.5%) with median age of 42 [IQR 34–47] years, 42 [34–46] for males, and 44 [33–47] for females (*P* = 0.471). Reasons for exclusion included lack of ICH (*n* = 765), subarachnoid hemorrhage (*n* = 78), traumatic cause of ICH (*n* = 57), ICH being recurrent (*n* = 38), patient records missing (*n* = 53), being initially treated in another hospital (*n* = 18), incorrect primary diagnosis of ICH (*n* = 8, 0.6%), and subdural hemorrhage (*n* = 5). Median length of acute hospital stay was 13 days (IQR 5–18). Baseline data of the study population appear in Table 1.

Hyperglycemia was the most frequent complication (50.8%), followed by hyponatremia (44.9%), hypopotassemia (32.0%), and hypernatremia (28.0%). Respiratory infection was the most common of infections (Table 2). Regarding gender differences, both respiratory infections (31.1% versus 16.7%, *P* = 0.003) and renal failure (24.4% versus 9.1%, *P* < 0.001) were more common among males than females. Prevalence of renal failure also increased with age (3.5%, 21.5%, and 21.2% in age groups of 16–29, 30–39, and 40–49 years, resp., *P* = 0.007). No clear correlation was found with other complications and sex or age.

Operative treatment was initiated in 111 (34.2%) patients, of which 102 (31.4%) underwent hematoma evacuation, 31 (9.5%) ventricular drainage, and 6 (1.8%) decompressive craniectomy. Respiratory infections, meningitis, hyperglycemia, and electrolyte disturbances with the exception of hyperpotassemia were significantly more common among those who received surgical treatment (Table 2). Furthermore, use of endotracheal intubation (108 [97.3%] versus 62 [29.0%], *P* < 0.001), tracheostomy (31 [27.9%] versus 7 [3.3%], *P* < 0.001), nasogastric tube (72 [64.9%] versus 45 [21.0%], *P* < 0.001), central vein catheter (58 [52.3%] versus 15 [7.0%], *P* < 0.001), and administration of antibiotics (82 [73.9%] versus 85 [39.7%]) were more frequent in patients undergoing surgical treatment. Cerebrospinal fluid sampling (38 [34.2%] versus 16 [7.5%], *P* < 0.001) and tracheal bacterial culture sampling (44 [39.6%] versus 17 [7.9%], *P* < 0.001) were also more frequently performed on patients receiving operative treatment, while there was no statistically significant difference with urine sampling (69 [62.2%] versus 153 [71.5%], *P* = 0.086).

During the acute phase, none of our patients suffered from ischemic heart event. Arrhythmias recorded included one brief ventricular fibrillation in a patient who was successfully resuscitated. One flutter and two first-ever atrial fibrillation cases were also diagnosed during the hospital stay.

Arrhythmia, renal failure, hyperglycemia, and hypernatremia associated with increased 3-month mortality in univariate analysis (Table 3). In the logistic regression model with known prognosticators and diabetes adjusted for, the only independent complication associated with mortality at 3 months was hyperglycemia, which increased the odds of dying nearly sixfold (odds ratio 5.90, 95% confidence interval 2.25–15.48) (Table 4). In the regression model with number of separate complications as the covariate of interest, 3 or more complications independently were associated with the risk of death by over sevenfold increase (7.76, 1.42–42.49, *P* = 0.018).

## 4. Discussion

Electrolyte and glucose disturbances, in particular hyperglycemia and hyponatremia, were common in the acute phase among young patients with ICH. After adjusting for confounders, only hyperglycemia was independently associated with increased mortality. Infections were as well rather frequent, but young patients seem to survive them. Importantly, increasing number of separate complications was gradually associated with increasing odds for death at 3 months. Our observations suggest that randomized trials aiming to investigate not only treatment of hyperglycemia in ICH but a holistic approach to treatment of medical complications to improve patient outcomes are warranted. Further, this finding underlines one of the mechanisms by which stroke unit care reduces mortality rates as prevention and quick treatment of complications in stroke patients are a hallmark of well-organized stroke units.

Hyperglycemia has been reported to worsen the outcome after ICH, but the exact underlying mechanisms are yet to be resolved [9, 10]. However, one study found no correlation between blood glucose and outcome after adjusting for ICH Score, and high blood glucose level was not associated with hematoma expansion or perihematomal edema [19]. Our and others' results indicate that better understanding of the role and mechanisms of hazard of hyperglycemia is needed. The optimal criteria and method for treatment of hyperglycemia are not solidly defined and should, therefore, be further investigated in a properly designed clinical trial [28, 29]. Hypoglycemia in our cases likely occurred due to excessive insulin treatment. The rates of hypoglycemia among stroke patients have not been previously reported. In contrast to prior findings with ischemic stroke [30], no association was observed with hypoglycemia and mortality, which may result due to our relatively small patient sample. Treatment of hyperglycemia should be adjusted more precisely in order to avoid hypoglycemia.

Electrolyte disturbances may arise after brain injury due to central nervous system having a major role in controlling sodium, potassium, and water homeostasis [31–33]. Potassium disturbances and outcome after ICH have not yet been studied. Prevalence of hyponatremia among ICH patients has been reported to be 15.6%, and it has been identified as a predictor of in-hospital mortality in a recent study [34]. Our findings, in contrast, suggest that hyponatremia or other individual electrolyte disturbances do not increase mortality in young ICH patients.

Stroke induces immunosuppression by cellular pathways, and infections are frequent after acute stroke [35]. The need for tracheostomy could potentially be predicted early in the course of ICH based on the admission GCS score, and early tracheostomy could decrease risks of prolonged endotracheal intubation [36]. In contrast to our findings with young ICH patients, respiratory infections are associated with poor outcome in general patients with ICH or ischemic stroke [5–8, 37, 38]. Reason for this remains unresolved, but possible explanations include lesser risk factors and comorbidities in young patients, more aggressive or active treatment strategies for infections, and more benign course of ICH [19].

Our results on the incidence of pneumonia among patients with ICH is in accordance with previous studies [5, 8]. Antibiotic treatment was initiated in half of our patients, which is markedly more than number of patients in which the infection site could be detected, suggesting that only a suspicion of an infection based on fever or mildly elevated inflammatory parameters frequently leads to administration of antimicrobial treatments. Alarming worldwide phenomenon of microbes becoming resistant to antibiotics raises the question whether the liberal use of antibiotics should be more restricted.

Patients with ICH are often immobilized due to impaired consciousness or motor hemiparesis increasing their risk for DVT and PE [8, 39]. Prior studies show that low-dose heparin treatment was not associated with increased hematoma growth after 48 hours of ICH [40]. However, it is yet to be decided whether more aggressive DVT surveillance is needed, since even in our series of young ICH patients, approximately 5% suffered from DVT or pulmonary embolism. However, neither of these showed association with higher mortality [41].

By our results, no myocardial ischemic in-hospital events were encountered and arrhythmias were considerably rare, which is in accordance with a previous report in nonselected ICH patients [22]. Although arrhythmias were more common in those dying within 3 months, no significant correlation with short-term mortality was observed after adjusting for confounders, which is probably due to the low number of patients (*n* = 4).

To our knowledge, renal failure has not previously been investigated in young patients with ICH, but in patients with ischemic stroke both low and high glomerular filtration rates have been reported to predict long-term mortality [24]. The relatively high number of renal failure cases observed in our study reflects the criteria we used but is rarer than in general ICH patients. A recent study found chronic kidney failure in nearly third of general ICH patients and it was associated with higher mortality [42]. Renal failure was associated with higher risk of death in our univariate analysis but not after adjusting for other prognosticators.

Our study has limitations. It is retrospective in nature, which may cause bias in the diagnoses of infectious complications. The exact interval by days from ICH onset to development of complication was not recorded. It is possible that in particular more infective and thromboembolic complications may have occurred after the acute hospital period. The ascertainment of PE and DVT depended on the physician pursuing the diagnostic work-up, and clinically silent DVTs have probably also gone undetected. Our patient cohort included only young patients, which makes the setting distinct compared to general ICH cohort, for example, with respect to lower mortality. Due to low number of deaths, some of our multivariable estimates may be instable and should be interpreted with caution. Nevertheless, we believe our relatively large number of patients compensates for the disadvantages of our study design.

## Figures and Tables

**Table 1:** Baseline characteristics of young ICH patients treated in Helsinki University Central Hospital between January, 2000, and March, 2010 (*N* = 325).

Factor	
Males	200 (59.5)
Age	42 (34–47)
Risk factors	
Hypertension	99 (30.5)
Diabetes	28 (8.6)
Hematoma characteristics	
Hematoma volume (mL)	11 (3–36)
Intraventricular hemorrhage	117 (36.0)
Infratentorial location	52 (16.0)
NIH Stroke Scale score at arrival	8 (2–19)
Etiology	
Hypertensive microangiopathy	84 (25.8)
Structural	84 (25.8)
Other	52 (16.0)
Unknown	105 (32.3)
Hematoma evacuation	102 (31.4)
Highest plasma glucose (mmol/L)	8.1 (6.5–10.1)
Lowest plasma glucose (mmol/L)	5.5 (4.9–6.2)
Highest plasma sodium (mmol/L)	143 (141–147)
Lowest plasma sodium (mmol/L)	137 (134–139)
Highest plasma potassium (mmol/L)	4.3 (4.0–4.6)
Lowest plasma potassium (mmol/L)	3.5 (3.2–3.7)
Highest plasma creatinine (*μ*mol/L)	75 (62–92)
Lowest plasma creatinine (*μ*mol/L)	59 (47–74)

Data are *n* (%) or median (interquartile range).

**Table 2:** Overview of in-hospital medical complications and conservative or operative treatment in 325 young patients with first-ever nontraumatic intracerebral hemorrhage.

Complication	All (*n* = 325)	Surgical treatment (*n* = 111)	Conservative treatment (*n* = 214)	*P* value
Any infection	110 (33.8)	57 (51.4)	53 (24.8)	<**0.001**
Sepsis	7 (2.2)	3 (2.7)	4 (1.9)	0.624
Respiratory infection	82 (25.2)	49 (44.1)	33 (15.4)	<**0.001**
Meningitis	2 (0.6)	2 (1.8)	0	**0.049**
Gastric infection	4 (1.2)	1 (0.9)	3 (1.4)	0.698
Urinary tract infection	40 (12.3)	14 (12.6)	26 (12.1)	0.904
Deep venous thrombosis	11 (3.4)	2 (1.8)	9 (4.2)	0.256
Pulmonary embolism	4 (1.2)	0	4 (1.9)	0.146
Arrhythmia	4 (1.2)	0	4 (1.9)	0.146
Renal failure	59 (18.2)	16 (14.4)	43 (20.1)	0.208
Hyperglycemia	165 (50.8)	88 (79.3)	77 (36.0)	<**0.001**
Hypoglycemia	15 (4.6)	6 (5.4)	9 (4.2)	0.625
Hyponatremia	146 (44.9)	66 (59.5)	80 (37.4)	<**0.001**
Hypernatremia	91 (28.0)	50 (45.0)	41 (19.2)	<**0.001**
Hypopotassemia	104 (32.0)	45 (40.5)	59 (27.6)	**0.022**
Hyperpotassemia	27 (8.3)	11 (9.9)	16 (7.5)	0.482

Data are *n* (%).

**Table 3:** Univariate analysis of in-hospital complications on 3-month mortality in young patients with intracerebral hemorrhage.

Complication	Alive (*n* = 270)	Dead (*n* = 55)	*P* value
Sepsis	5 (1.9)	2 (3.6)	0.406
Respiratory infection	63 (23.3)	19 (34.5)	0.081
Meningitis	2 (0.7)	0	0.522
Gastric infection	4 (1.5)	0	0.364
Urinary tract infection	39 (14.4)	1 (1.8)	**0.009**
Deep venous thrombosis	10 (3.7)	1 (1.8)	0.481
Pulmonary embolism	3 (1.1)	1 (1.8)	0.665
Arrhythmia	1 (0.4)	3 (5.5)	**0.002**
Renal failure	43 (15.9)	16 (29.1)	**0.021**
Hypoglycemia	10 (3.7)	5 (9.1)	0.083
Hyperglycemia	122 (45.2)	43 (78.2)	<**0.001**
Hyponatremia	117 (43.7)	29 (53.7)	0.176
Hypernatremia	66 (24.6)	25 (46.3)	**0.001**
Hypopotassemia	81 (30.2)	23 (42.6)	0.076
Hyperpotassemia	19 (7.1)	8 (14.8)	0.063
Number of complications			
0	64 (23.7)	2 (3.6)	<**0.001**
1	64 (23.7)	1 (1.8)	
2	50 (18.5)	16 (29.1)	
3 or more	92 (34.1)	36 (65.5)	

Data are *n* (%).

**Table 4:** Logistic regression analysis of hyperglycemia and 3-month mortality in young patients with intracerebral hemorrhage.

Factor	OR (95% CI)	*P* value
Female	1.78 (0.71–4.49)	0.220
Age group		
16–29	1	N.A.
30–39	0.04 (0.01–0.25)	**0.001**
40–49	0.27 (0.09–0.81)	**0.020**
Hematoma volume (mL)		
0–30	1	N.A.
30–60	1.75 (0.61–5.01)	0.296
>60	2.97 (0.80–10.97)	0.103
NIH Stroke Scale, per point	1.18 (1.13–1.23)	<**0.001**
Intraventricular hemorrhage	1.44 (0.57–3.60)	0.441
Infratentorial location	0.40 (0.12–1.24)	0.109
Hematoma evacuation	0.07 (0.02–0.20)	<**0.001**
Arrhythmia	1.23 (0.04–37.93)	0.908
Renal failure	1.99 (0.76–5.21)	0.159
Hyperglycemia	5.90 (2.25–15.48)	<**0.001**
Hypernatremia	1.33 (0.50–3.54)	0.563
Diabetes	1.54 (0.40–6.01)	0.533

OR, odds ratio; CI, confidence interval.
